# Supplementary material for: Immune Responses in Checkpoint Myocarditis Across Heart, Blood, and Tumor
Source: bioRxiv. 2023 Nov 29:2023.09.15.557794. Originally published 2023 Sep 18. Preprint. [Version 2] doi: 10.1101/2023.09.15.557794 (PMC10542127; doi:10.1101/2023.09.15.557794)

bioRxiv preprint doi: https://doi.org/10.1101/2023.09.15.557794; this version posted November 29, 2023. The copyright holder for this preprint (which was not certified by peer review) is the author/funder, who has granted bioRxiv a license to display the preprint in perpetuity. It is made available under aCC-BY 4.0 International license.

Supplementary Figure Legends

Supplementary Figure 1. Cell lineages in heart and blood defined by scRNA-seq. a, Dot plot showing top marker genes for each lineage in the heart. Dot size represents the percent of cells in the lineage with non-zero expression of a given gene. Color indicates scaled expression across lineages. **b,** Stacked bar plots showing the composition of major cell lineages, colored by data source ("MGH" refers to data generated in this study; "Sanger" refers to public heart atlas data)[29]. **c,** UMAP embedding of cell density plot displaying the relative proportion of cells from irMyocarditis cases and controls. **d,** Blood samples and timepoints collected for the study. Red patient labels denote samples from patients with fatal irMyocarditis. Two ICI-treated patients who did not develop irMyocarditis (SIC_31, SIC_109) are denoted with purple dots. **e,** Dot plot showing top marker genes for each lineage in the blood. Dot size represents the percent of cells in the lineage with non-zero expression of a given gene. Color indicates scaled expression across lineages. **f,** Lineage proportions (y-axis) versus serum troponin (x-axis) for pre-corticosteroid irMyocarditis samples. Unadjusted linear model p-values are shown. **Related to Figure 1.**

Supplementary Figure 2. Lymphoid cells in irMyocarditis tissue. a, UMAP embedding of a cell density plot showing the contribution of cells from irMyocarditis cases and controls projected on the UMAP of T and NK cells derived from heart scRNA-seq data. **b,** Stacked bar plots showing the per-subset cellular composition per donor of each pre-corticosteroid or unenriched control sample. Red patient labels denote samples from patients with fatal irMyocarditis. **c, d** The 33 expanded TCR-β sequences (> 0.5% of TCR-β repertoire) from patient SIC_264 in T/NK UMAP space, color coded by whether the cell was found prior to ("pre-corticosteroid", red) or after ("post-corticosteroid", blue) administration of corticosteroids and second-line immunosuppression. Data shown by TCR-β clone (**c**) and in aggregate of all clones (**d**). **e,** Feature plot using color to indicate gene expression (logCPM) levels of *STMN1* projected onto the T/NK UMAP embedding. Cell numbers and percentages represent gene expression across all T/NK cells. **Related to Figure 2.**

Supplementary Figure 3. Expanded T-cell receptor β (TCR-β) sequences in irMyocarditis tissue. a, A boxplot showing the relative proportion of cells that recovered a TCR-β CDR3 sequence from marked areas of irMyocarditis (red) and control (blue) tissue; p = 0.0002, T-test (via Adaptive Biotechnologies). **b,** Expanded TCR-β CDR3 (left) and total TCR-β CDR3 sequences (right) recovered on a per patient basis from both scRNA-seq and bulk TCR-β CDR3 sequencing. **c,** Expanded TCR-β CDR3 from bulk sequencing (left) and scRNA-seq data from matched blood (right) in patients with irMyocarditis. **d,** Within each tissue type in each patient ("control", "tumor", or

bioRxiv preprint doi: https://doi.org/10.1101/2023.09.15.557794; this version posted November 29, 2023. The copyright holder for this preprint (which was not certified by peer review) is the author/funder, who has granted bioRxiv a license to display the preprint in perpetuity. It is made available under aCC-BY 4.0 International license.

"irMyocarditis"), the frequency of each TCR-β clone is plotted on a per-patient basis and labeled by the pathological designation of the macroscopically dissected regions (SIC_17: Active; SIC_136: Borderline; SIC_3: Healing; SIC_175: Healing). Each point represents a TCR-β clone. The y-axis represents the proportion of a given TCR-β clone in the patient's control tissue repertoire, and the x-axis represents the proportion of the TCR-β clone in their tumor TCR-β repertoire (left column) or irMyocarditis TCR-β repertoire (right column). Points are pseudocolored to represent a TCR-β clone that was expanded (> 0.5% of the heart or tumor repertoire) and enriched (Fisher's exact test FDR < 5% compared to control) in heart (green), tumor (purple), both tissues (red), or neither tissue (grey). **e**, The frequency of each expanded TCR-β clone in heart and tumor tissue was calculated and then normalized by dividing by the frequency of the same clone in control tissue. Normalized TCR-β clone frequencies for heart (x-axis) and tumor tissue (y-axis) are plotted. Each plot shows the enriched TCR-β clones within each donor projected onto the aggregate data across all donors (**Figure 3c-d**). Individual data points from a given patient, representing TCR-β clones from that patient contributing to the aggregate plot, are colored by location of enrichment – heart (green), tumor (purple), both tissues (red), or not enriched (grey). In **a,** boxes represent the median (line) and interquartile range (IQR) with whiskers extending to the remainder of the distribution, no more than 1.5x IQR, with dots representing individual samples. Throughout the figure, red patient labels denote cases of fatal irMyocarditis. **Related to Figure 3.**

Supplementary Figure 4. CD8 T and NK cells in blood. a, Feature plots using color to indicate gene expression (logCPM) levels of the indicated marker genes, number of unique genes expressed by each cells (bottom left) and percent mitochondrial genes (bottom right), projected onto the blood CD8 T/NK UMAP embedding. Cell numbers and percentages represent gene expression across all blood CD8 T/NK cells. **b,** Blood CD8 T/NK UMAP highlighting circulating cells (in red) that express a TCR-β sequence found to be expanded in irMyocarditis hearts (combined scRNA-seq and bulk TCR-β sequencing data). **c,** A volcano plot showing the results of a logistic regression model investigating the likelihood of a cell in a given CD8 T/NK cell subset in blood containing a TCR-β CDR3 sequence that was expanded in heart tissue. Red points denote cell subsets with statistically significant sharing (FDR < 0.05, likelihood-ratio test) **d**, Cells in blood for which the same TCR-β was expanded in paired irMyocarditis heart and blood sample from the same patient are shown in red on CD8 T/NK blood UMAP embeddings. Each patient with paired heart and blood samples is shown. **e,** UMAP of heart T and NK cells highlighting cells that express expanded TCR-β CDR3 sequences that were found in blood on a per-patient basis. **f,** Feature plots using color to indicate gene expression (logCPM) levels of the indicated genes projected onto the heart T and NK UMAP embedding. Cell numbers and percentages represent gene expression across all heart T

bioRxiv preprint doi: https://doi.org/10.1101/2023.09.15.557794; this version posted November 29, 2023. The copyright holder for this preprint (which was not certified by peer review) is the author/funder, who has granted bioRxiv a license to display the preprint in perpetuity. It is made available under aCC-BY 4.0 International license.

and NK cells. In **e**, error bars represent 95% confidence intervals. In **f** and **g**, red patient labels denote cases of fatal irMyocarditis. **Related to Figure 3.**

Supplementary Figure 5. CD4 T cells in blood. a, UMAP embedding of 33,313 CD4 T cells from blood, colored by the six defined cell subsets labeled on the right. Cell subset number was assigned according to the absolute number of cells detected per subset. **b,** Dot plot showing top marker genes for each CD4 T cell subset in the blood. Dot size represents the percent of cells in the subset with non-zero expression of a given gene. Color indicates scaled expression across subsets. **c,** Left: feature plots using color to indicate surface protein levels (logCPM) of CD45RA and CD45RO protein (as determined by CITE-seq) projected onto the blood CD4 T cell UMAP embedding. Right: feature plots using color to indicate gene expression levels (logCPM) of the indicated genes projected onto the blood CD4 T cell UMAP embedding. Cell numbers and percentages represent gene expression across all blood CD4 T cells. **Related to Figure 3.**

Supplementary Figure 6. MNP populations in heart. a, Embedding of cell density plot showing the contribution of cells from irMyocarditis versus control samples projected on the UMAP of MNP cells derived from heart scRNA-seq data. **b**, Stacked bar chart depicting the relative contributions of cells in each MNP subset (colored coded on the right) on a per donor basis from each pre-corticosteroid irMyocarditis or unenriched control sample. **c**, Comparison of CD1c+ cell density measured by immunohistochemical staining of control heart sections (left column) versus whole slides from irMyocarditis heart sections (right column); p = 0.11, one-sided T-test. **d**, Comparison of CD1c+ cell density measured by immunohistochemical staining of control heart sections (left column) versus regions of inflammation in irMyocarditis heart sections (right column); p = 0.053, one-sided T-test. **e**, Feature plots using color to indicate gene expression (logCPM) levels of the indicated genes projected onto the blood MNP UMAP embedding. Cell numbers and percentages represent gene expression across all blood MNP cells. **f**, Feature plots showing percent mitochondrial genes (top panel), or number of unique genes expressed by each cell (bottom panel) on the UMAP of MNP cells derived from blood scRNA-seq data. **Related to Figure 4.**

Supplementary Figure 7. Non-immune populations in irMyocarditis heart tissue. a, Feature plots using color to indicate marker gene expression (logCPM) levels of the indicated genes projected onto the heart non-immune UMAP embedding. Cell numbers and percentages represent gene expression across all heart non-immune cells. **b**, Feature plots showing percent mitochondrial genes (left panel), or number of genes expressed by each cell (right panel) on the UMAP of non-immune cells derived from heart scRNA-seq data. **c**, UMAP embedding of cell density plot showing the contribution of cells from irMyocarditis versus control samples projected on the UMAP of non-immune cells

bioRxiv preprint doi: https://doi.org/10.1101/2023.09.15.557794; this version posted November 29, 2023. The copyright holder for this preprint (which was not certified by peer review) is the author/funder, who has granted bioRxiv a license to display the preprint in perpetuity. It is made available under aCC-BY 4.0 International license.

derived from heart scRNA-seq data. **d**, Stacked bar chart depicting the relative contributions of cells in each non-immune cell subset (color coded to the right) from each pre-corticosteroid irMyocarditis or unenriched control sample. **Related to Figure 5.**

Supplementary Figure 8. Sorting strategy for myocardial samples. Pseudocolor plots showing the applied sequential gating strategy to sort live cells for downstream scRNAseq from a representative myocardial sample. Numbers indicate the percentage within the indicated gate. $DAPI^{-}CD235a^{-}$ cells were collected for downstream analysis. **Related to Methods.**

bioRxiv preprint doi: https://doi.org/10.1101/2023.09.15.557794; this version posted November 29, 2023. The copyright holder for this preprint (which was not certified by peer review) is the author/funder, who has granted bioRxiv a license to display the preprint in perpetuity. It is made available under aCC-BY 4.0 International license.

Supplementary figure 1

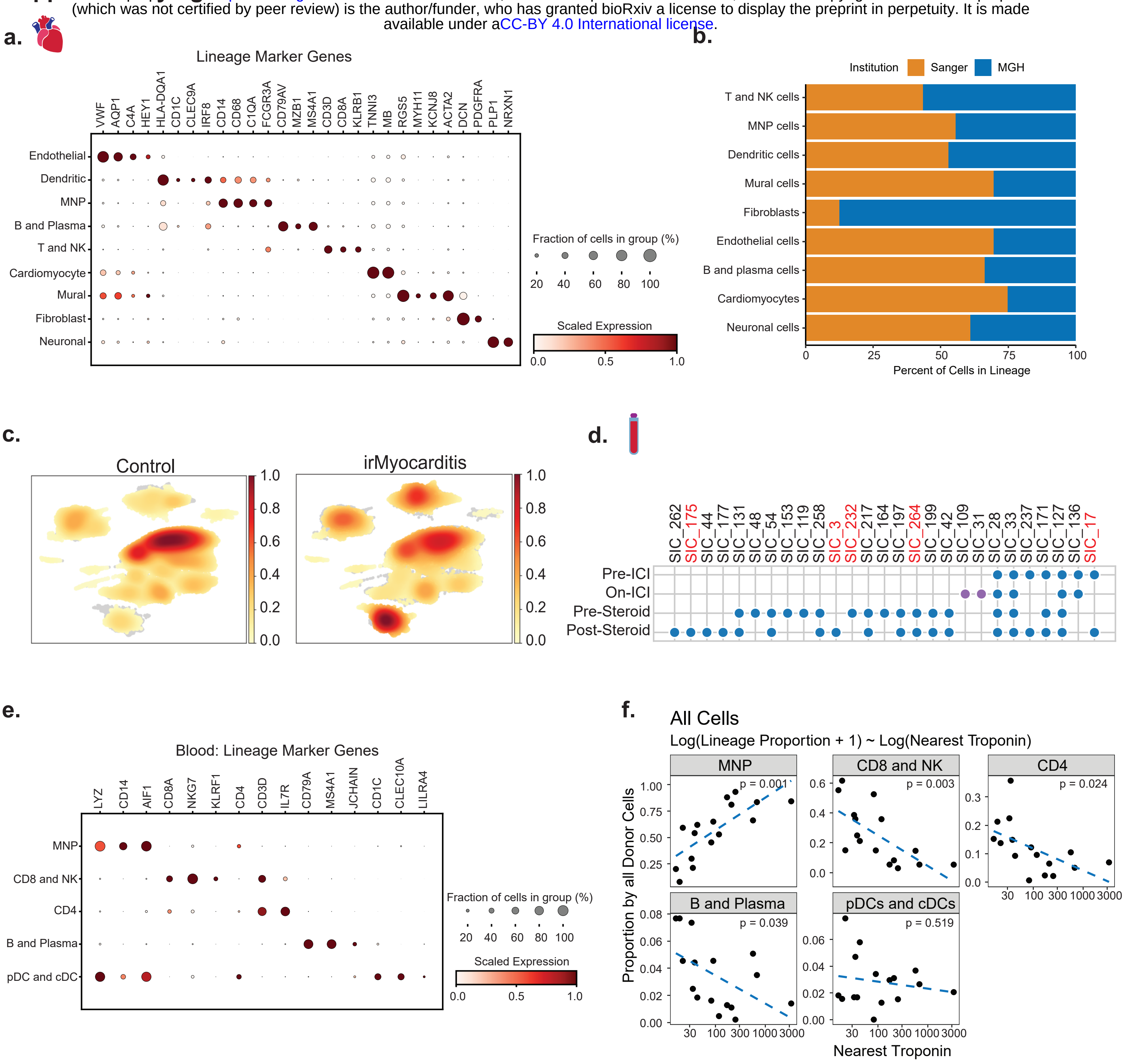

bioRxiv preprint doi: https://doi.org/10.1101/2023.09.15.557794; this version posted November 29, 2023. The copyright holder for this preprint (which was not certified by peer review) is the author/funder, who has granted bioRxiv a license to display the preprint in perpetuity. It is made available under aCC-BY 4.0 International license.

Supplementary figure 2

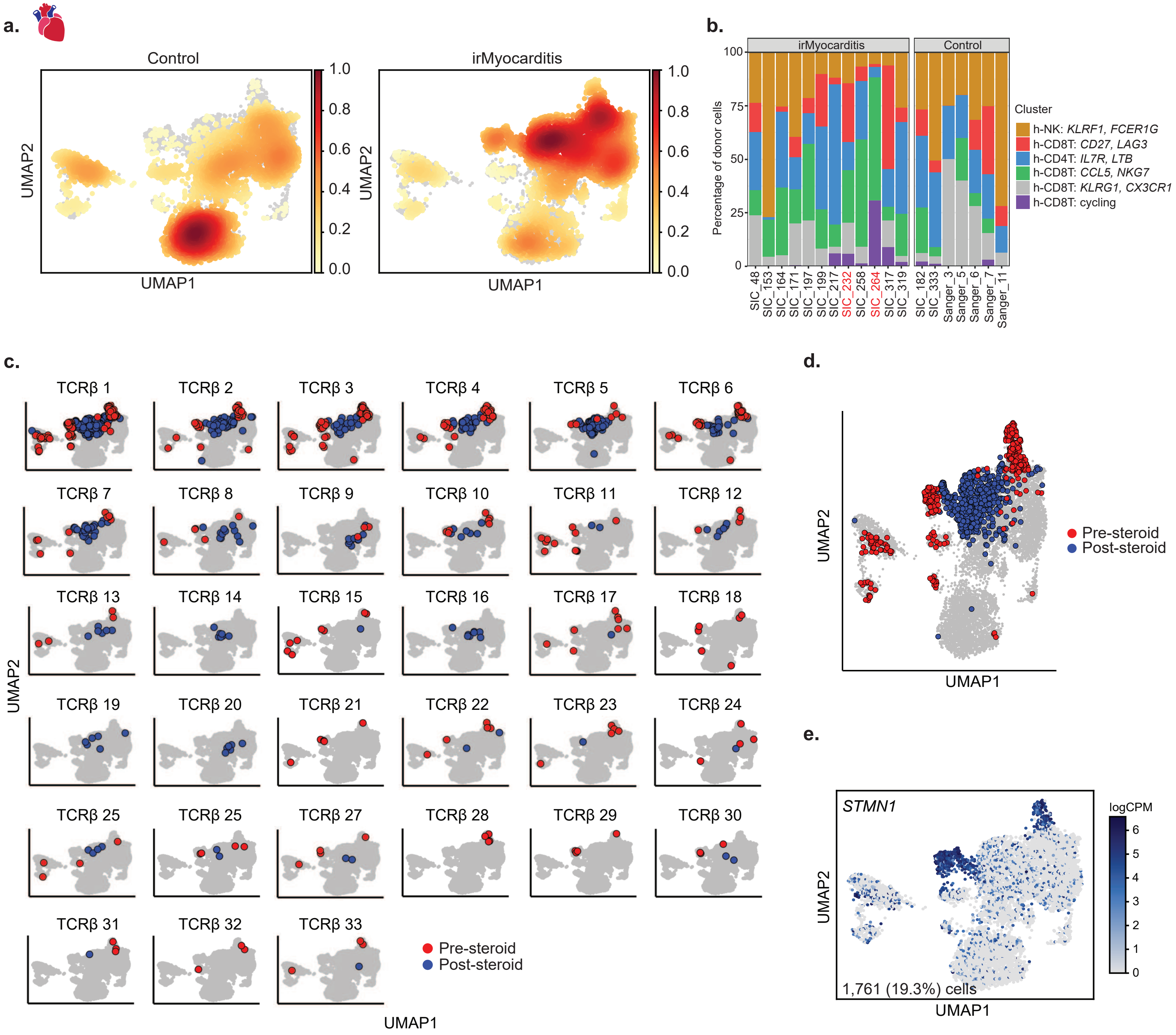

Supplementary figure 3

bioRxiv preprint doi: https://doi.org/10.1101/2023.09.15.557794; this version posted November 29, 2023. The copyright holder for this preprint (which was not certified by peer review) is the author/funder, who has granted bioRxiv a license to display the preprint in perpetuity. It is made available under aCC-BY 4.0 International license.

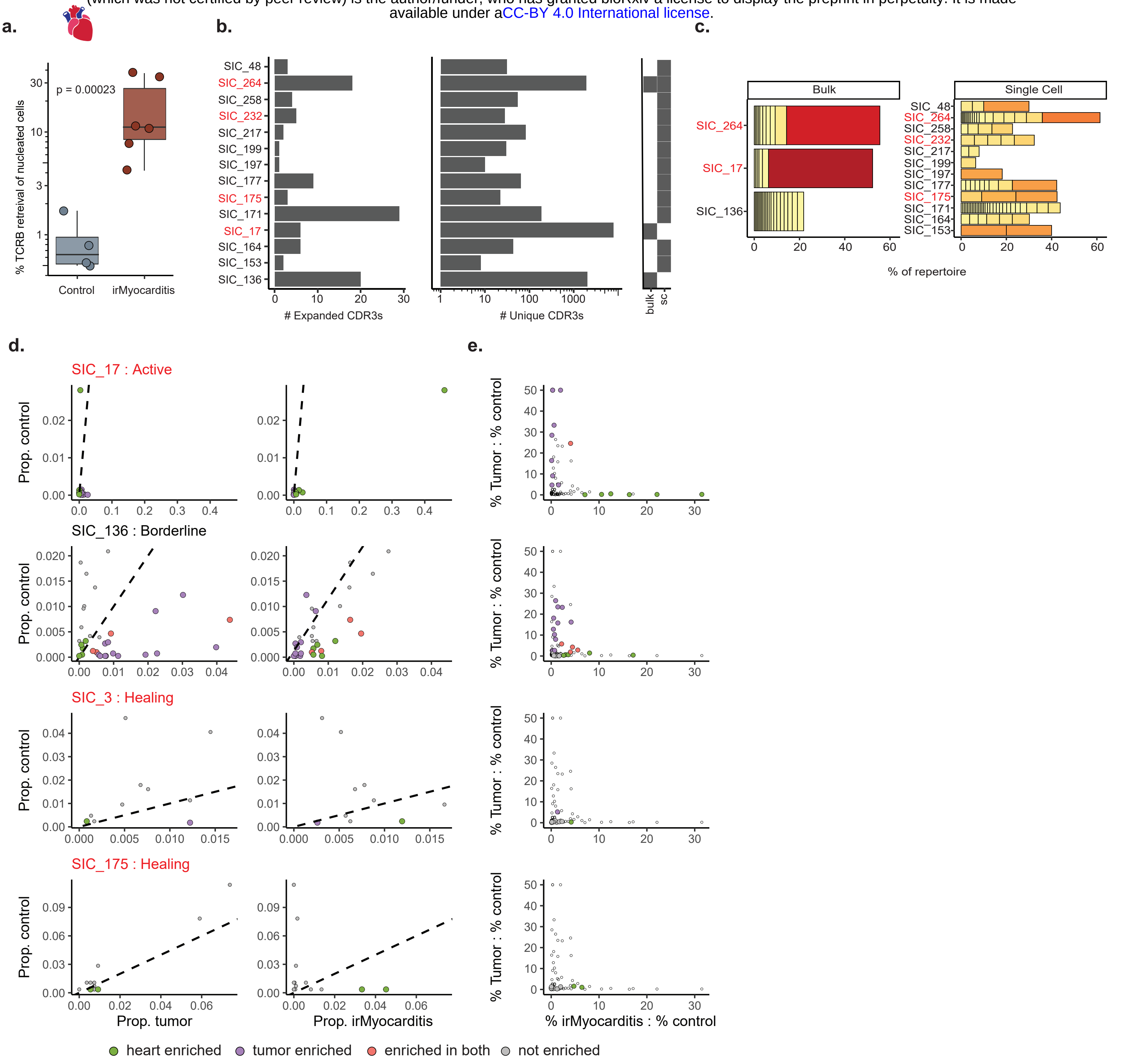

bioRxiv preprint doi: https://doi.org/10.1101/2023.09.15.557794; this version posted November 29, 2023. The copyright holder for this preprint (which was not certified by peer review) is the author/funder, who has granted bioRxiv a license to display the preprint in perpetuity. It is made available under aCC-BY 4.0 International license.

Supplementary figure 4

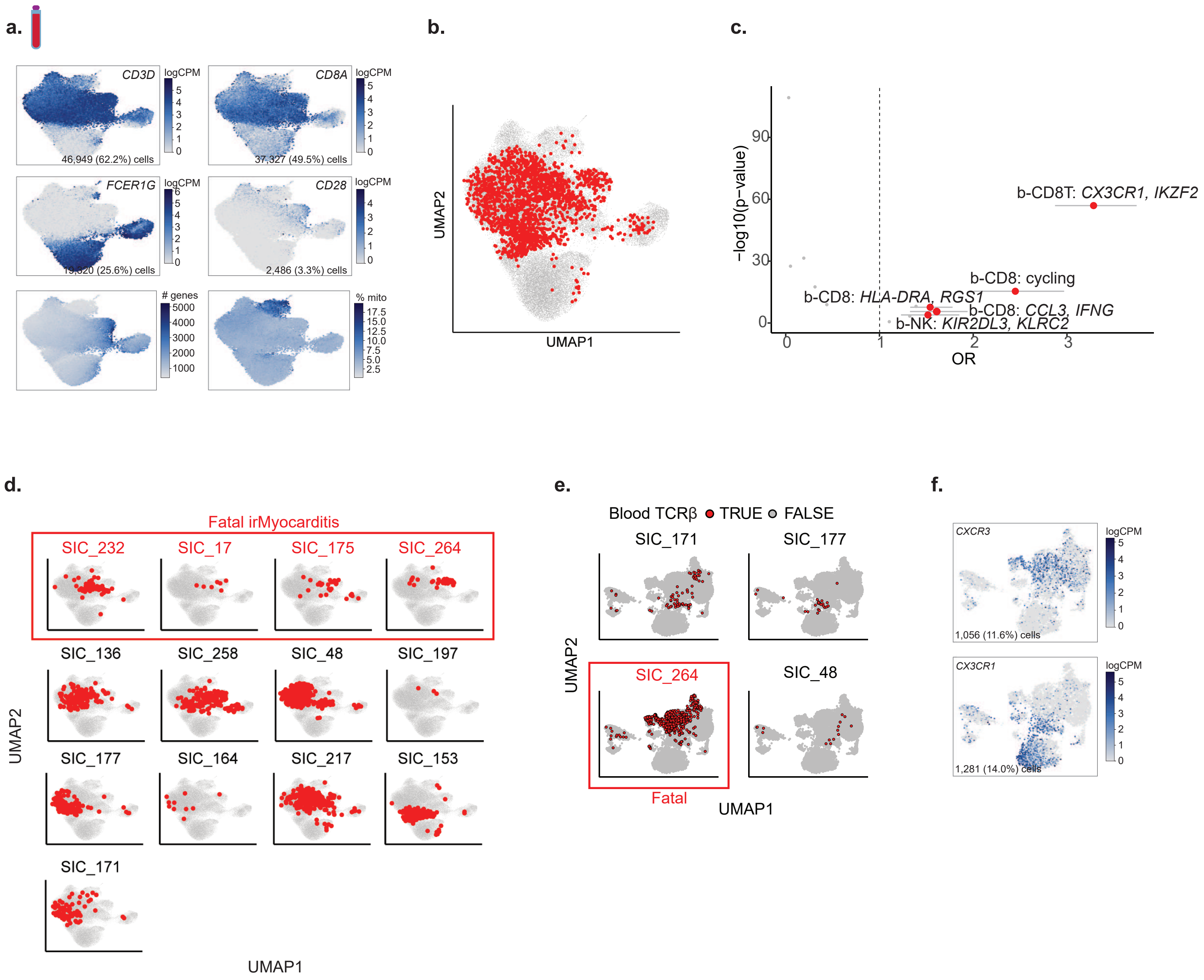

bioRxiv preprint doi: https://doi.org/10.1101/2023.09.15.557794; this version posted November 29, 2023. The copyright holder for this preprint (which was not certified by peer review) is the author/funder, who has granted bioRxiv a license to display the preprint in perpetuity. It is made available under aCC-BY 4.0 International license.

Supplementary figure 5

a.

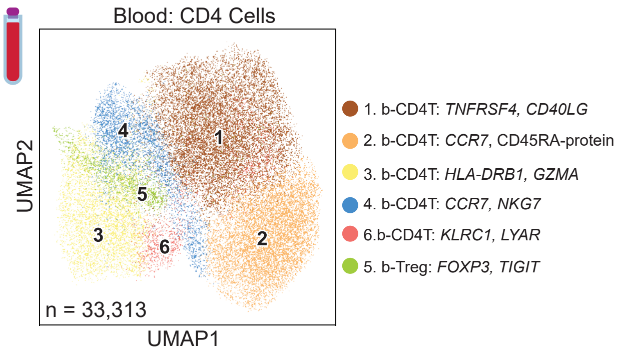

b.

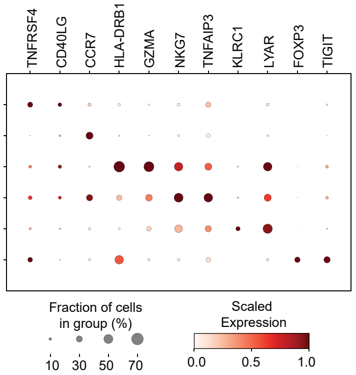

c.

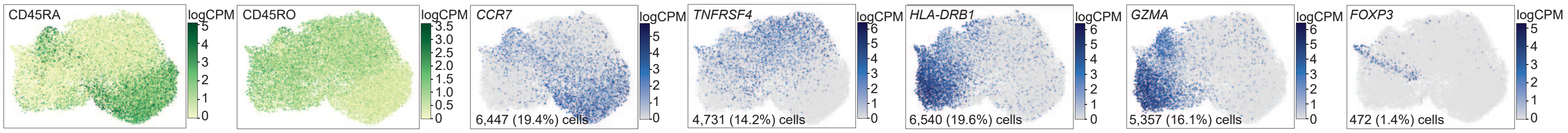

bioRxiv preprint doi: https://doi.org/10.1101/2023.09.15.557794; this version posted November 29, 2023. The copyright holder for this preprint (which was not certified by peer review) is the author/funder, who has granted bioRxiv a license to display the preprint in perpetuity. It is made available under aCC-BY 4.0 International license.

Supplementary figure 6

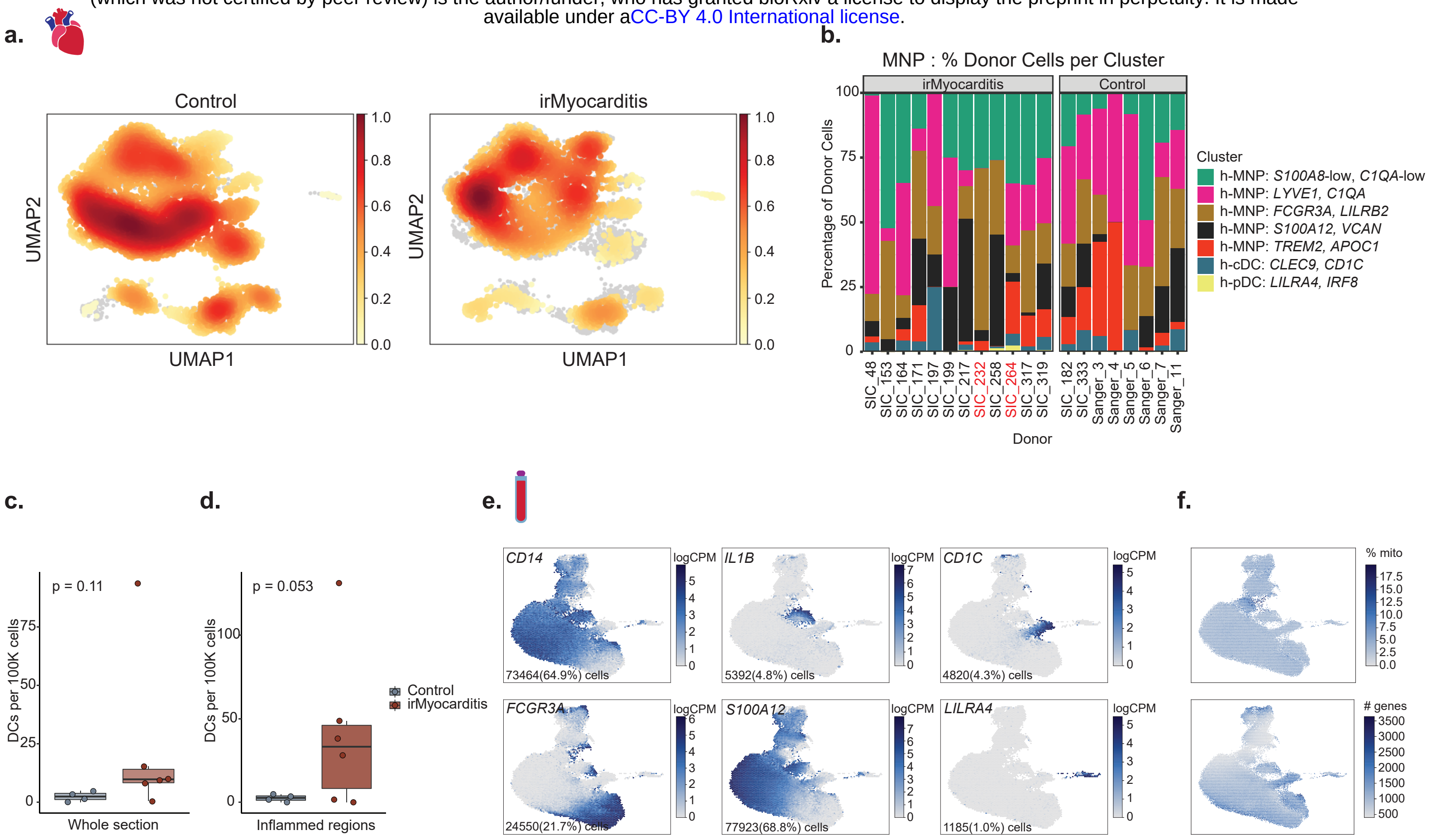

bioRxiv preprint doi: https://doi.org/10.1101/2023.09.15.557794; this version posted November 29, 2023. The copyright holder for this preprint (which was not certified by peer review) is the author/funder, who has granted bioRxiv a license to display the preprint in perpetuity. It is made available under aCC-BY 4.0 International license.

Supplementary figure 7

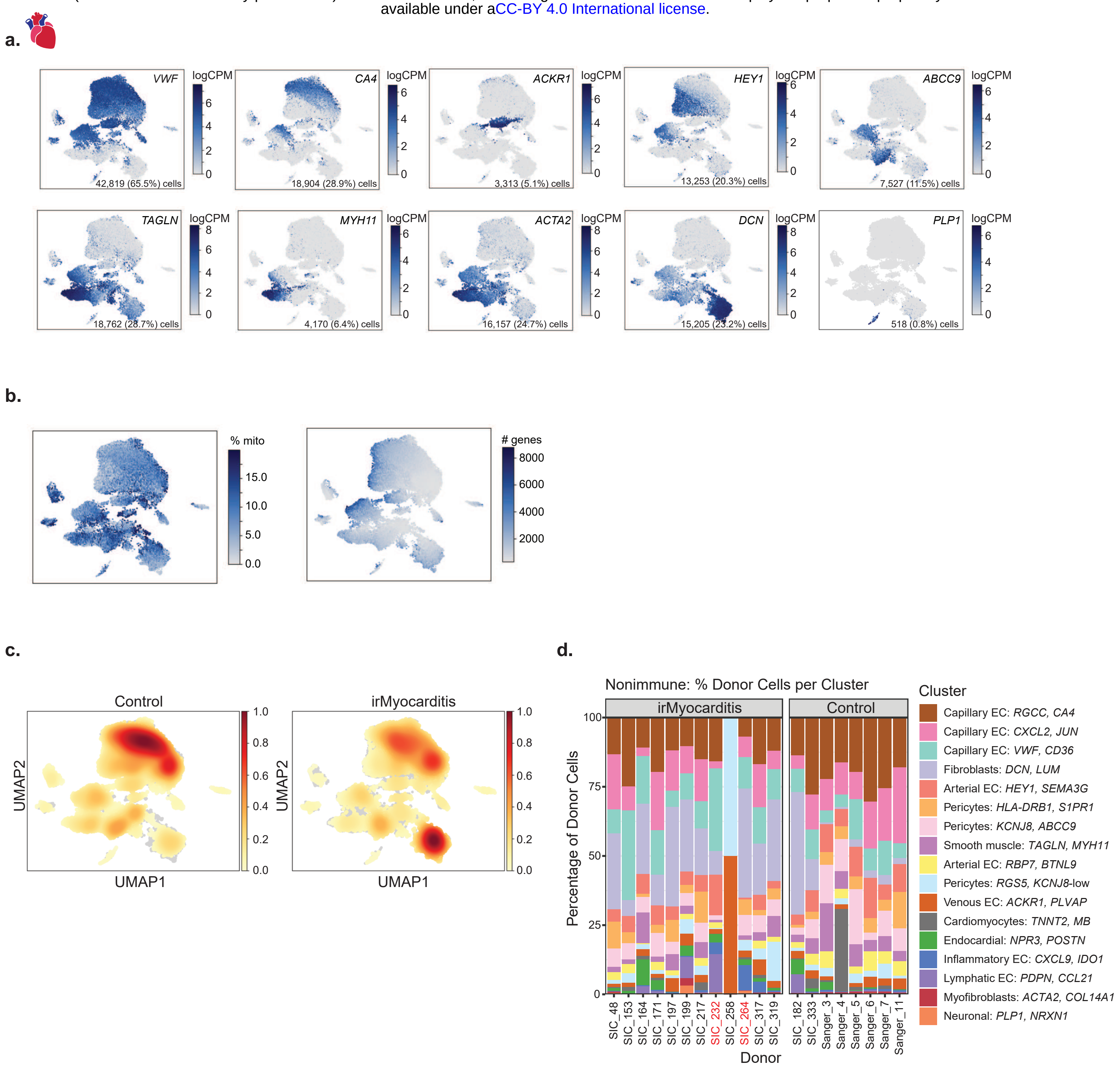

bioRxiv preprint doi: https://doi.org/10.1101/2023.09.15.557794; this version posted November 29, 2023. The copyright holder for this preprint (which was not certified by peer review) is the author/funder, who has granted bioRxiv a license to display the preprint in perpetuity. It is made available under aCC-BY 4.0 International license.

Supplementary figure 8

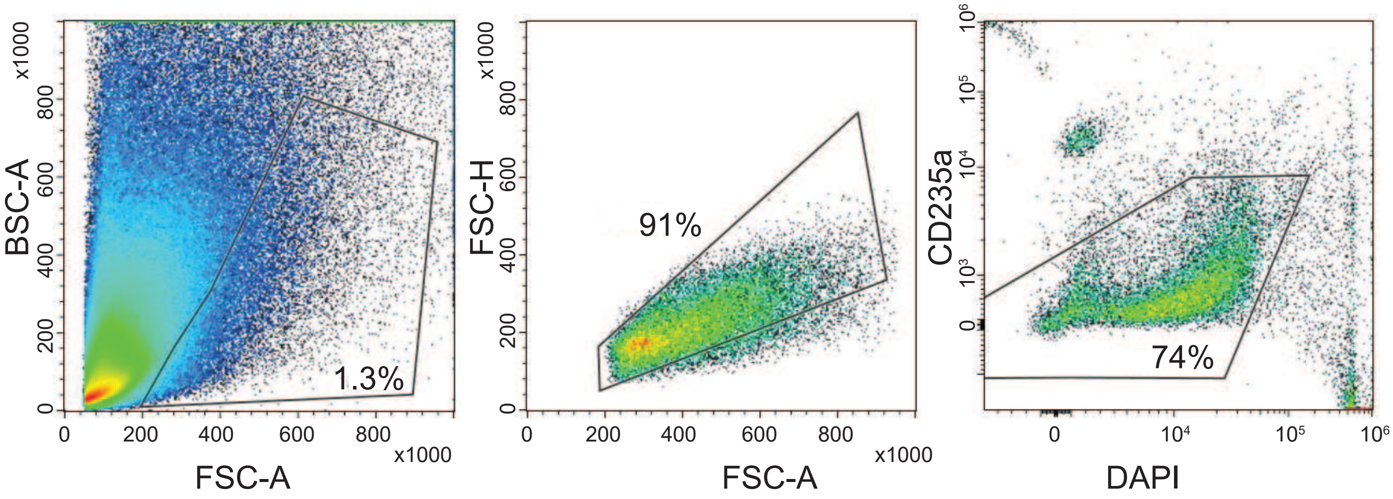

Supplement: Supplement 1 [file NIHPP2023.09.15.557794v2-supplement-1.pdf]
