# Supplementary material for: Immune Responses in Checkpoint Myocarditis Across Heart, Blood, and Tumor
Source: bioRxiv. 2023 Sep 18:2023.09.15.557794. Preprint. [Version 1] doi: 10.1101/2023.09.15.557794 (PMC10542127; doi:10.1101/2023.09.15.557794)

bioRxiv preprint doi: https://doi.org/10.1101/2023.09.15.557794; this version posted September 18, 2023. The copyright holder for this preprint (which was not certified by peer review) is the author/funder, who has granted bioRxiv a license to display the preprint in perpetuity. It is made available under aCC-BY 4.0 International license.

Supplementary figure 1

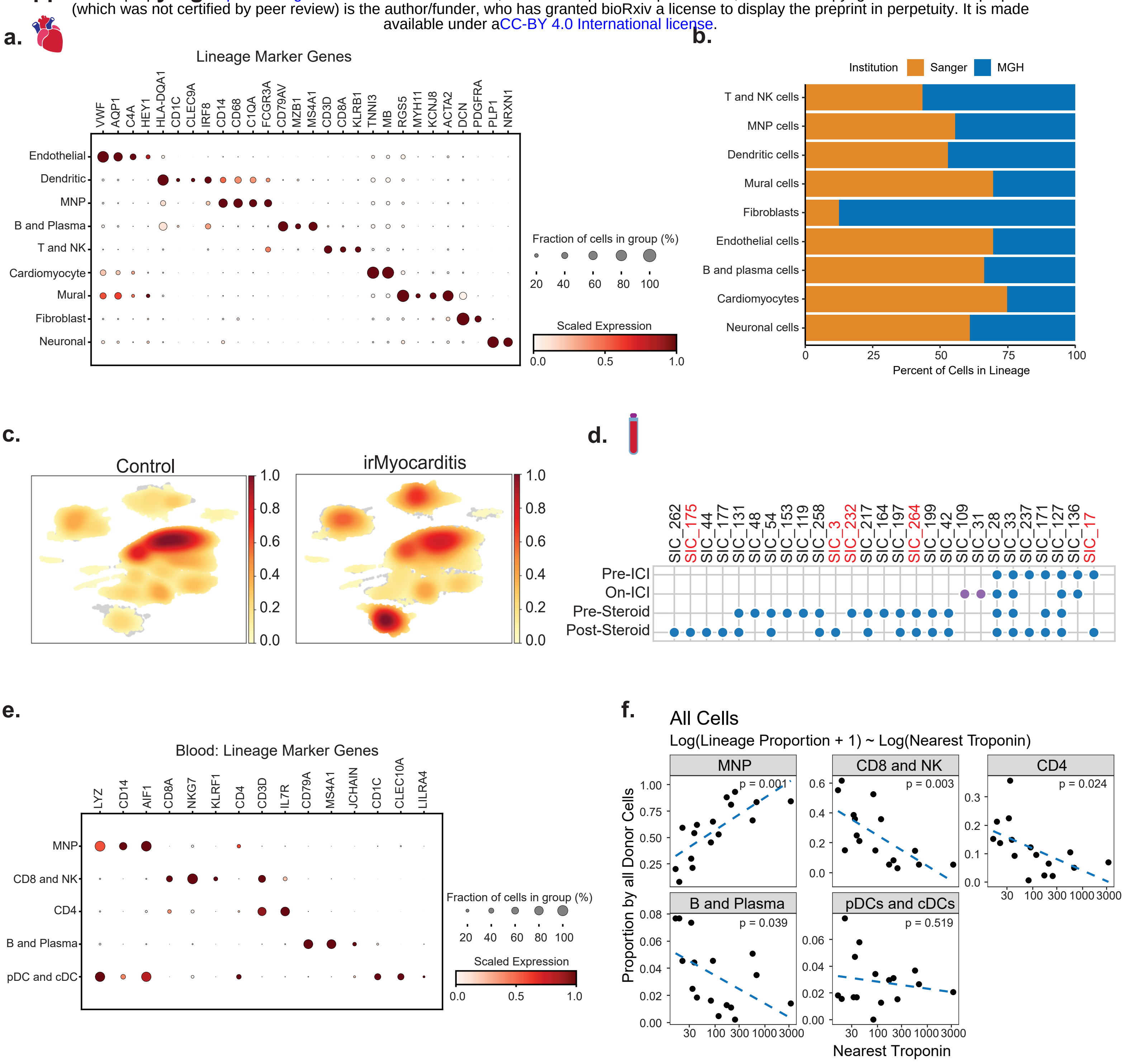

bioRxiv preprint doi: https://doi.org/10.1101/2023.09.15.557794; this version posted September 18, 2023. The copyright holder for this preprint (which was not certified by peer review) is the author/funder, who has granted bioRxiv a license to display the preprint in perpetuity. It is made available under aCC-BY 4.0 International license.

Supplementary figure 2

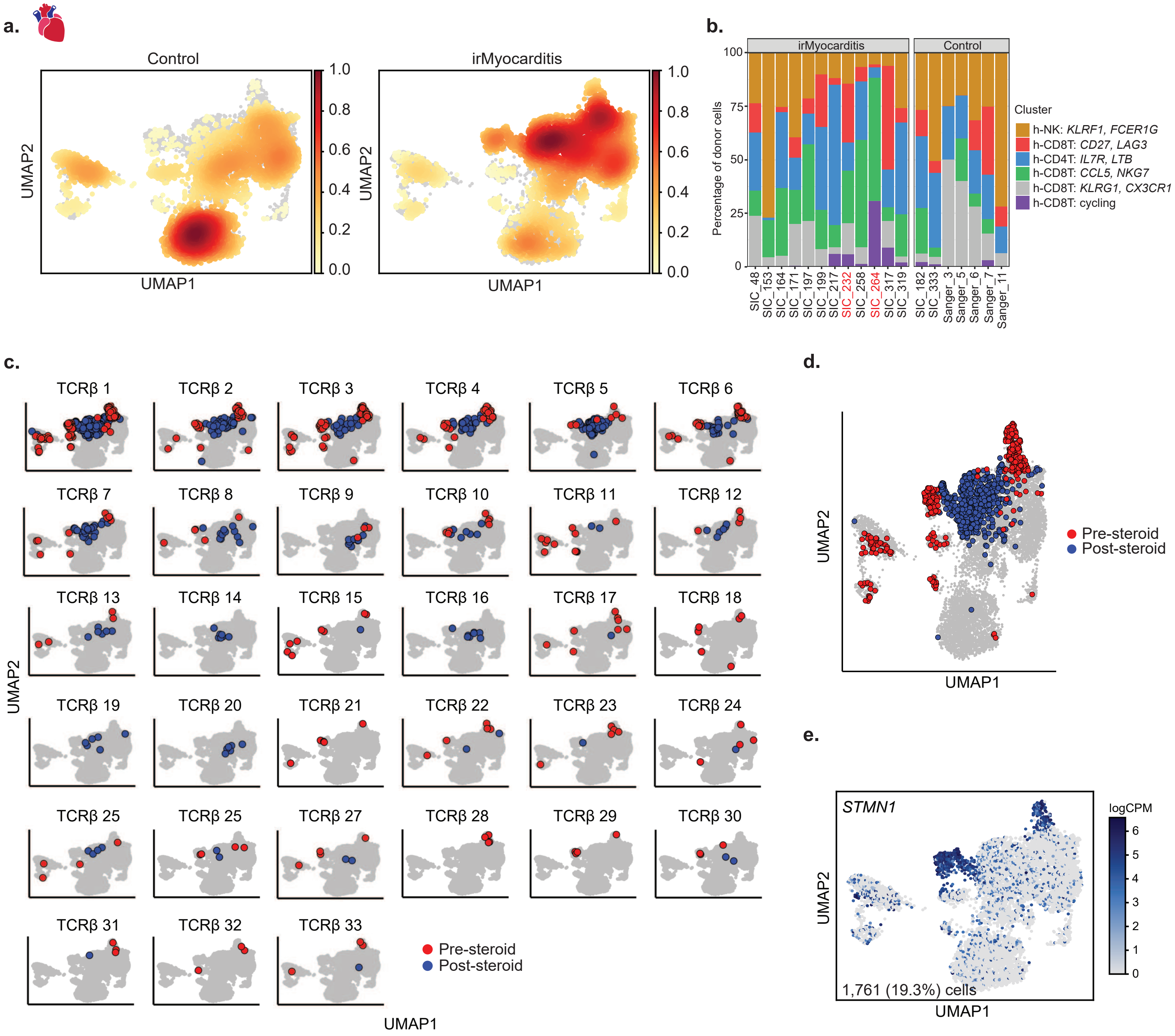

bioRxiv preprint doi: https://doi.org/10.1101/2023.09.15.557794; this version posted September 18, 2023. The copyright holder for this preprint (which was not certified by peer review) is the author/funder, who has granted bioRxiv a license to display the preprint in perpetuity. It is made available under aCC-BY 4.0 International license.

Supplementary figure 3

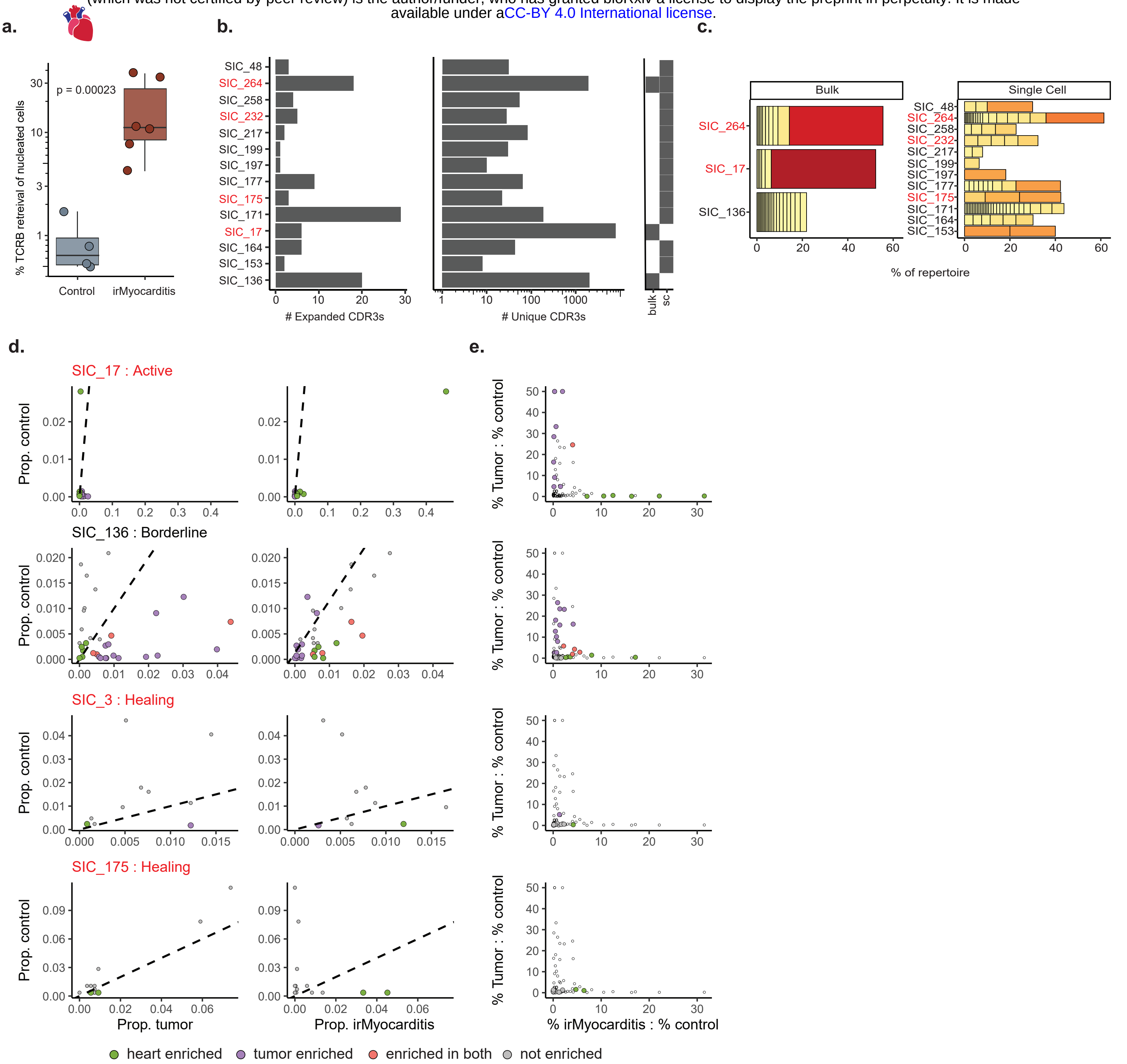

bioRxiv preprint doi: https://doi.org/10.1101/2023.09.15.557794; this version posted September 18, 2023. The copyright holder for this preprint (which was not certified by peer review) is the author/funder, who has granted bioRxiv a license to display the preprint in perpetuity. It is made available under aCC-BY 4.0 International license.

Supplementary figure 4

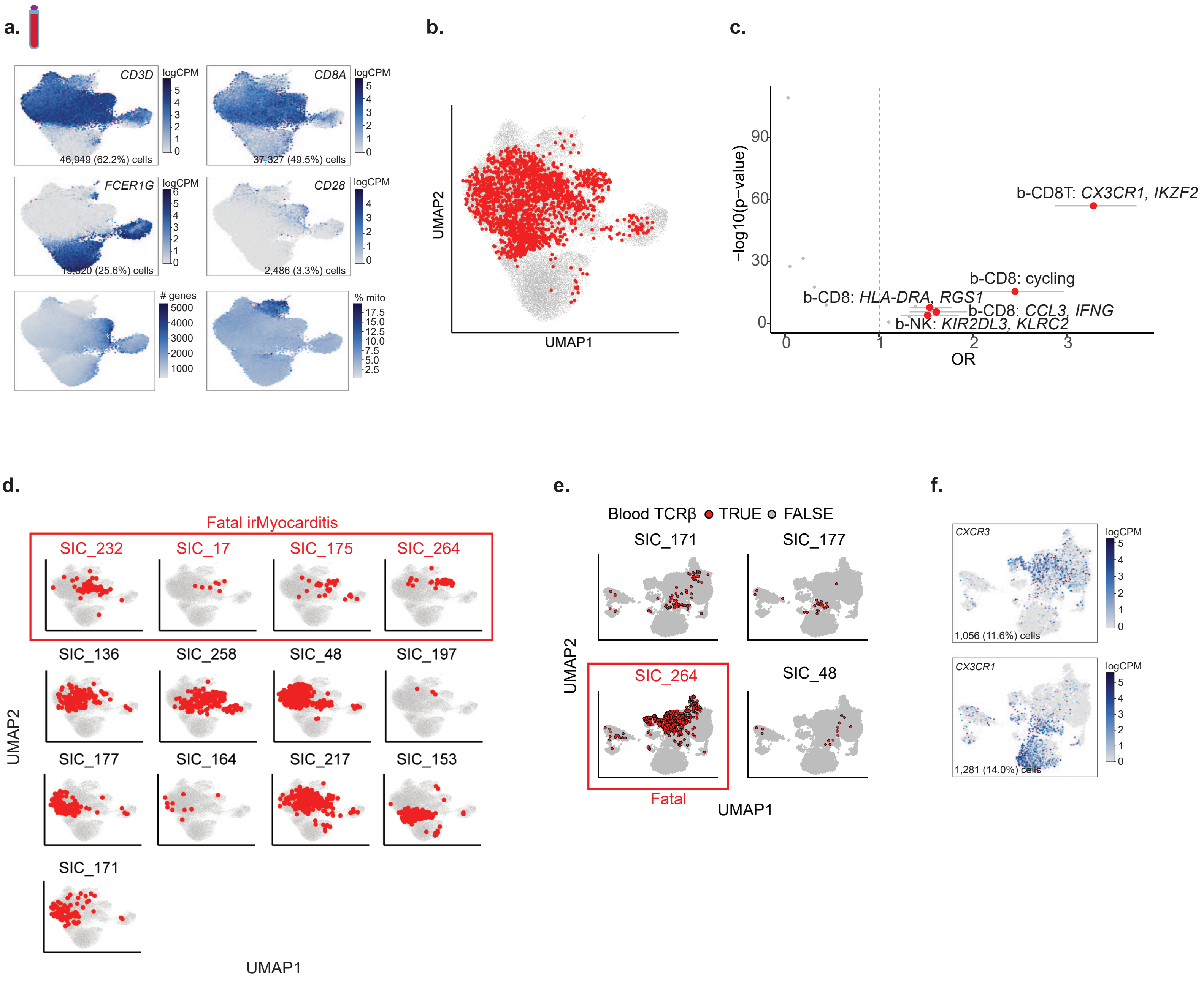

bioRxiv preprint doi: https://doi.org/10.1101/2023.09.15.557794; this version posted September 18, 2023. The copyright holder for this preprint (which was not certified by peer review) is the author/funder, who has granted bioRxiv a license to display the preprint in perpetuity. It is made available under aCC-BY 4.0 International license.

Supplementary figure 5

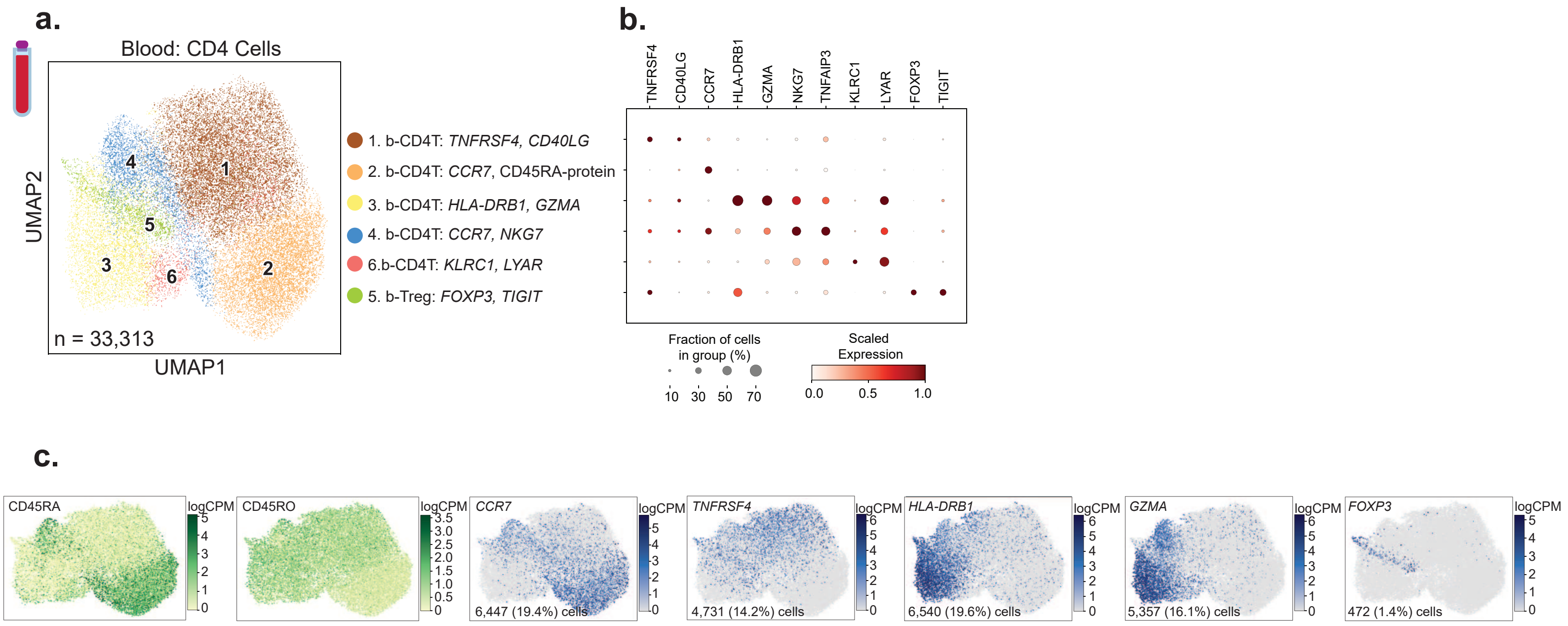

bioRxiv preprint doi: https://doi.org/10.1101/2023.09.15.557794; this version posted September 18, 2023. The copyright holder for this preprint (which was not certified by peer review) is the author/funder, who has granted bioRxiv a license to display the preprint in perpetuity. It is made available under aCC-BY 4.0 International license.

Supplementary figure 6

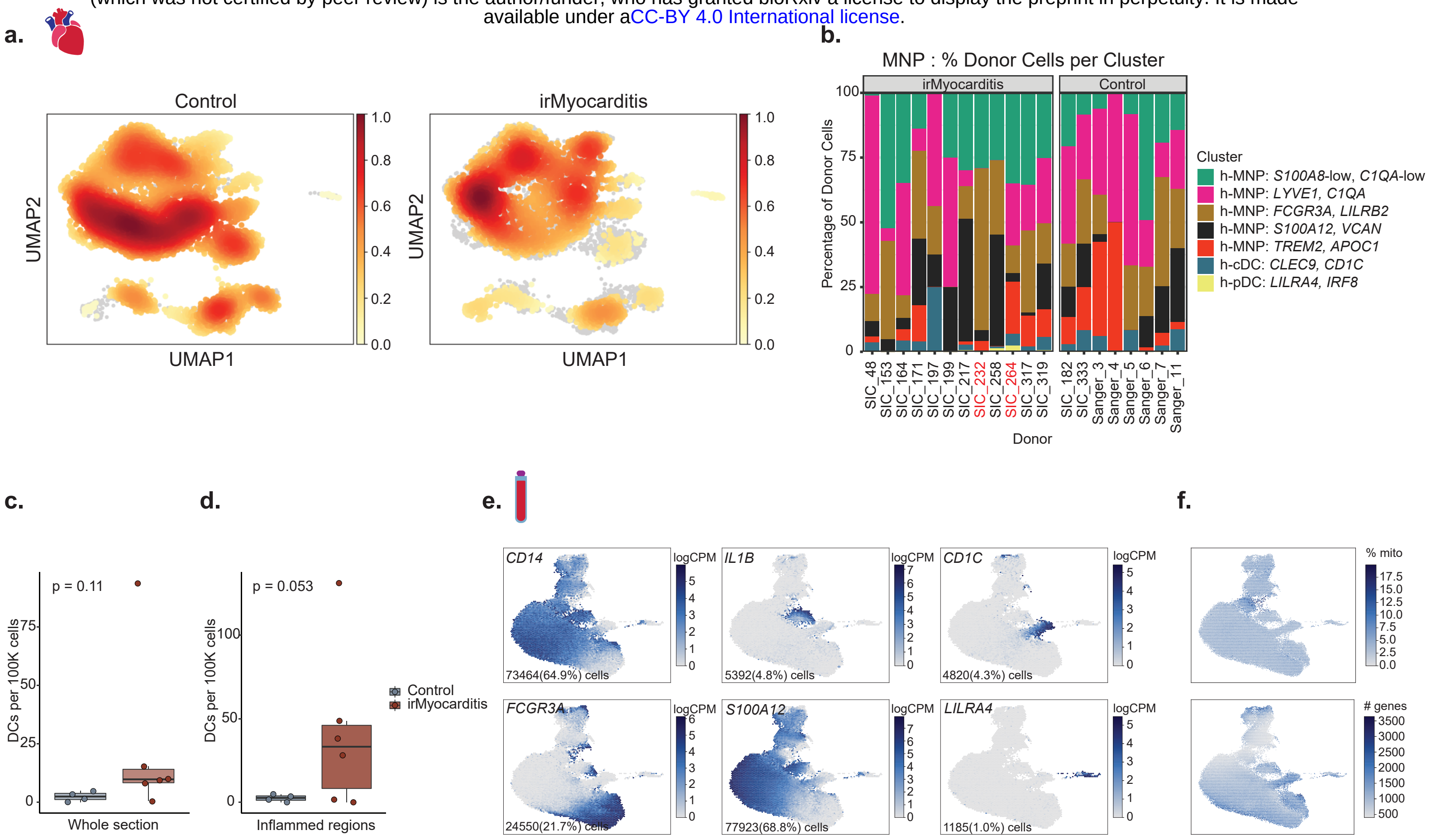

bioRxiv preprint doi: https://doi.org/10.1101/2023.09.15.557794; this version posted September 18, 2023. The copyright holder for this preprint (which was not certified by peer review) is the author/funder, who has granted bioRxiv a license to display the preprint in perpetuity. It is made available under aCC-BY 4.0 International license.

Supplementary figure 7

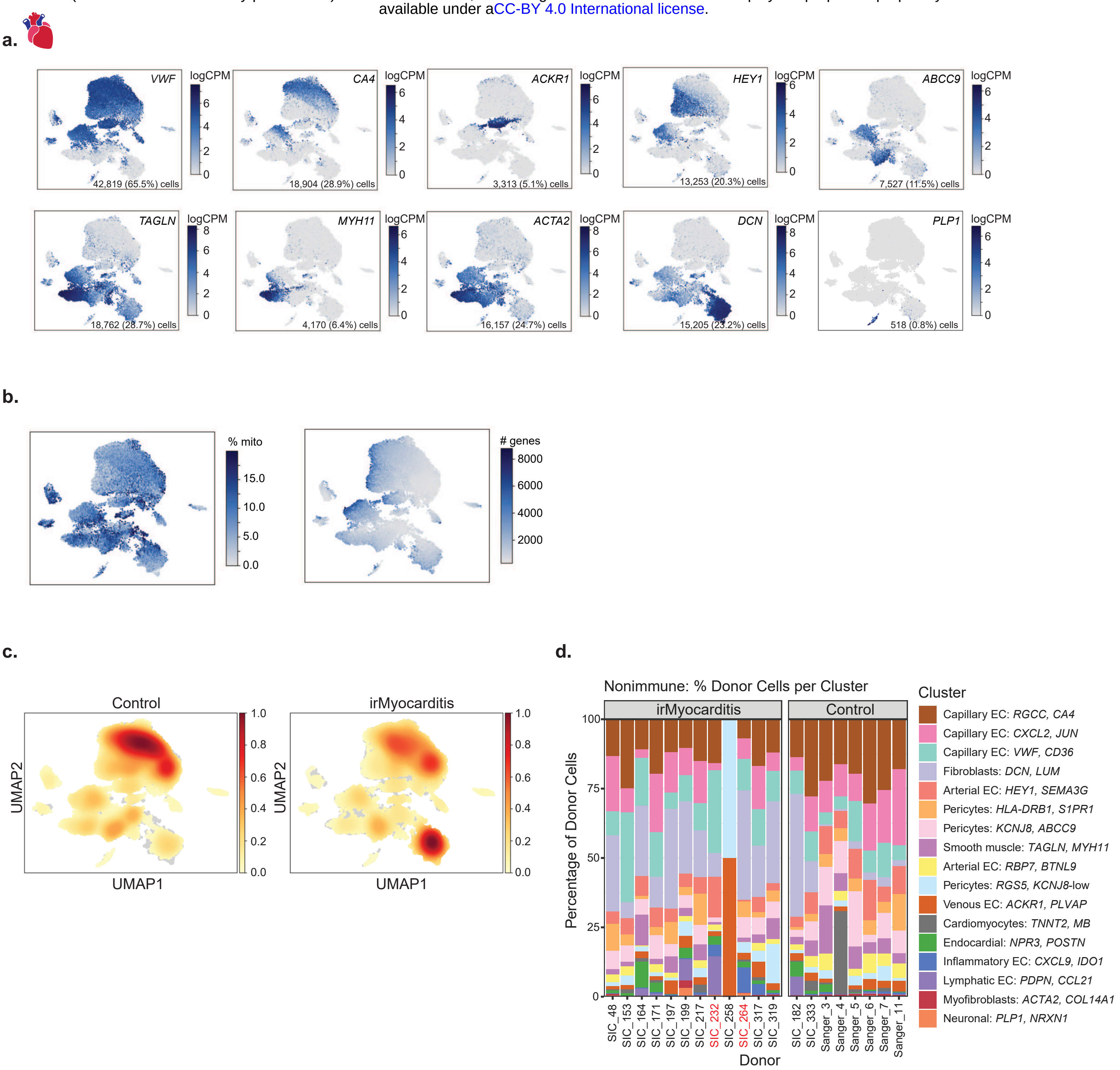

bioRxiv preprint doi: https://doi.org/10.1101/2023.09.15.557794; this version posted September 18, 2023. The copyright holder for this preprint (which was not certified by peer review) is the author/funder, who has granted bioRxiv a license to display the preprint in perpetuity. It is made available under aCC-BY 4.0 International license.

Supplementary figure 8

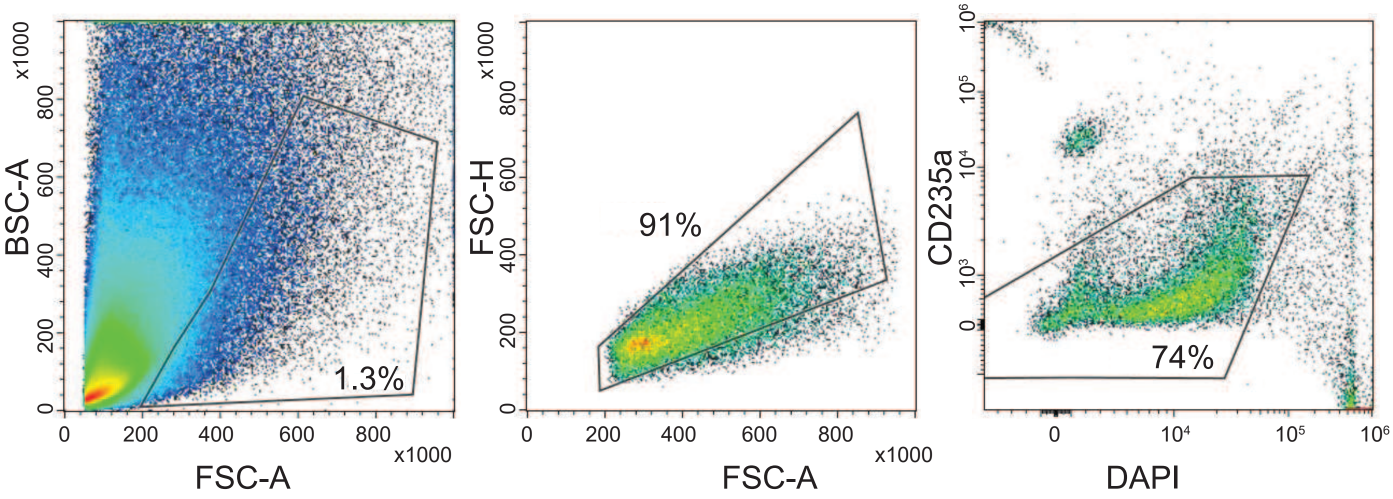

Supplement: Supplementary file 1 [file NIHPP2023.09.15.557794V1-supplement-1.pdf]
